# Down‐regulation of human long non‐coding RNA LINC01187 is associated with nephropathies

**DOI:** 10.1111/jcmm.17014

**Published:** 2023-04-13

**Authors:** Theodora Manolakou, Valeria Kaltezioti, Niki Prakoura, Panagiotis Kavvadas, Simone Reichelt‐Wurm, Harikleia Gakiopoulou, Miriam Banas, Bernhard Banas, Maja T. Lindenmeyer, Clemens D. Cohen, Peter Boor, Sonja Djudjaj, Dimitrios T. Boumpas, Christos Chatziantoniou, Aristidis Charonis, Panagiotis K. Politis

**Affiliations:** ^1^ Center for Basic Research Biomedical Research Foundation of the Academy of Athens Athens Greece; ^2^ Center for Clinical Experimental Surgery and Translational Research Biomedical Research Foundation of the Academy of Athens Athens Greece; ^3^ School of Medicine National and Kapodistrian University of Athens Greece; ^4^ Batiment Recherche Tenon Hospital Paris France; ^5^ Department of Nephrology University Hospital Regensburg Regensburg Germany; ^6^ 1st Department of Pathology School of Medicine National & Kapodistrian University of Athens Athens Greece; ^7^ III. Department of Medicine University Medical Center Hamburg‐Eppendorf Hamburg Germany; ^8^ Nephrological Center Medical Clinic and Policlinic IV University Hospital of Munich Munich Germany; ^9^ Institute of Pathology RWTH Aachen University Hospital Aachen Germany; ^10^ Department of Nephrology and Immunology RWTH Aachen University Hospital Aachen Germany; ^11^ Rheumatology and Clinical Immunology Unit 4th Department of Internal Medicine Attikon University Hospital Joint Rheumatology Program National and Kapodistrian University of Athens Medical School Athens Greece; ^12^ Faculty of Medicine Sorbonne University Paris France; ^13^ Institut National de la Santé et de la Recherche Médicale (INSERM) Unité Mixte de Recherche Scientifique 1155 Tenon Hospital Paris France; ^14^ University Research Institute of Maternal and Child Health and Precision Medicine Athens Greece

**Keywords:** apoptosis, *Gm12121*, in situ hybridization, *LINC01187*, long non‐coding RNAs, renal diseases

## Abstract

Chronic kidney diseases affect a substantial percentage of the adult population worldwide. This observation emphasizes the need for novel insights into the molecular mechanisms that control the onset and progression of renal diseases. Recent advances in genomics have uncovered a previously unanticipated link between the non‐coding genome and human kidney diseases. Here we screened and analysed long non‐coding RNAs (lncRNAs) previously identified in mouse kidneys by genome‐wide transcriptomic analysis, for conservation in humans and differential expression in renal tissue from healthy and diseased individuals. Our data suggest that *LINC01187* is strongly down‐regulated in human kidney tissues of patients with diabetic nephropathy and rapidly progressive glomerulonephritis, as well as in murine models of kidney diseases, including unilateral ureteral obstruction, nephrotoxic serum‐induced glomerulonephritis and ischemia/reperfusion. Interestingly, *LINC01187* overexpression in human kidney cells *in vitro* inhibits cell death indicating an anti‐apoptotic function. Collectively, these data suggest a negative association of *LINC01187* expression with renal diseases implying a potential protective role.

## 1 INTRODUCTION

Kidney and common systemic diseases such as diabetes, hypertension and systemic lupus erythematosus lead to compromised functional integrity of the kidneys, culminating in chronic kidney disease (CKD).^1^, ^2^, ^3^, ^4^, ^5^, ^6^ CKD, defined as the decrease and gradual loss of filtering function, is one of the most prevalent health problems.^7^, ^8^, ^9^, ^10^ For most of its course, CKD is asymptomatic as the manifestation of the disease appears only when there is pronounced deterioration of the renal function. At this late stage, therapeutic intervention is of limited effectiveness.^11^ Therefore, it is crucial to elucidate the molecular mechanisms underlying renal pathophysiology in order to develop new therapeutic insights and alleviate the symptoms of CKD.

Recent advances in genomic technologies^12^, ^13^, ^14^, ^15^ have begun to unveil the complex molecular networks involved in human renal pathologies. Interestingly, these approaches have linked differential expression of long non‐coding RNAs (lncRNAs), a newly discovered class of regulatory RNAs, to the onset and progression of renal diseases. LncRNAs are defined as RNAs larger than 200 nucleotides long.^16^, ^17^ They are mostly transcribed by RNA polymerase II and can be modified by 5′ capping, 3′ polyadenylation and splicing. In many cases, their expression is regulated in a tissue‐specific and spatio‐temporal manner.^18^, ^19^, ^20^ RNA sequence conservation of lncRNAs among species is observed at a much lower level as compared to protein‐coding transcripts.^21^, ^22^ However, sequence conservation at the promoter sites is comparable to protein‐coding genes, especially for functionally important lncRNAs.^23^, ^24^, ^25^ Thus, lncRNAs constitute a critical new class of biomolecules for understanding cell physiology, organ homeostasis and pathological processes.

Herein, we report that *LINC01187*, a conserved lncRNA between mouse and human, is critically involved in nephropathies. We found that *LINC01187* is strongly reduced in human renal pathologies, including diabetic nephropathy (DN) and rapidly progressive glomerulonephritis (RPGN), as well as in murine models for kidney diseases including unilateral ureteral obstruction (UUO), nephrotoxic serum‐induced glomerulonephritis (NTS‐induced GN) and ischemia/reperfusion (I/R). Importantly, forced expression of *LINC01187* in human kidney cells inhibits cell death suggesting an anti‐apoptotic function. Together these data propose a negative association of *LINC01187* expression with renal pathologies implying a protective role.

## 2 MATERIALS AND METHODS

### 2.1 Human samples

**FIGURE 1:**
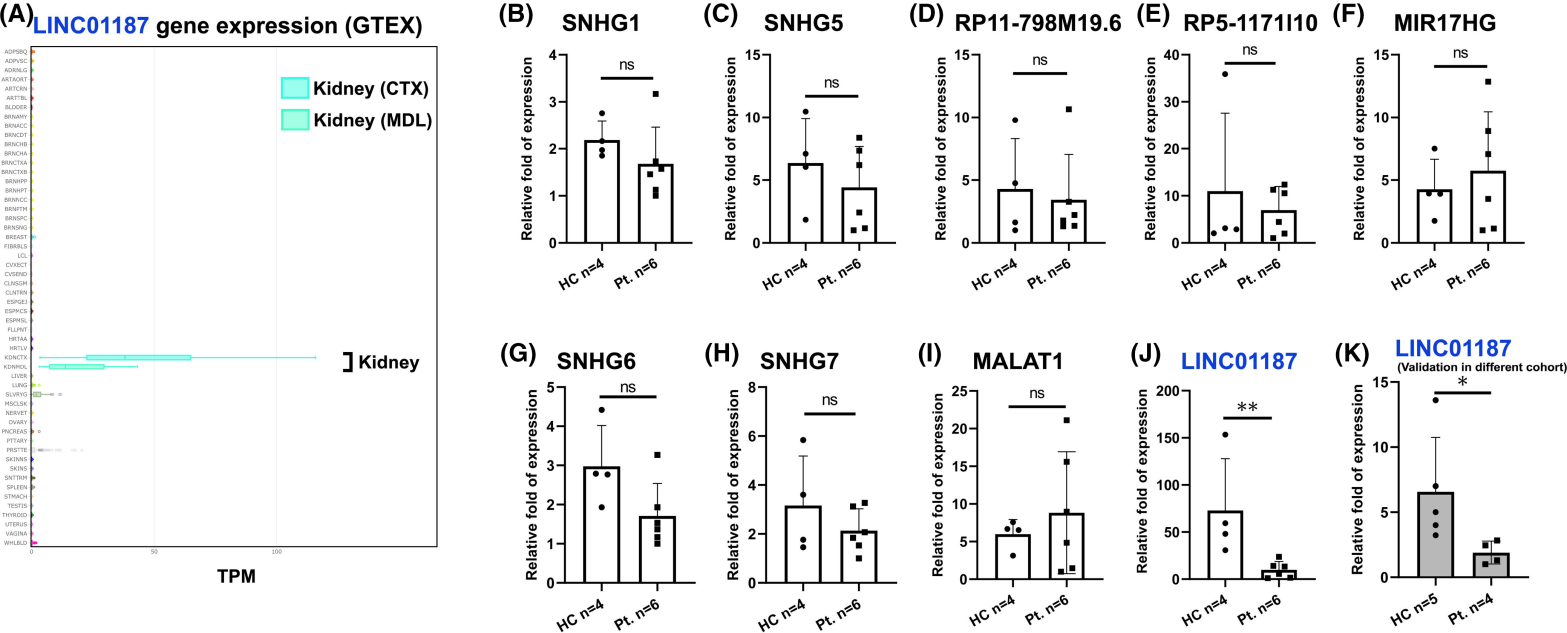
Expression analysis of selected lncRNAs in human renal biopsies. (A) Human Genotype‐Tissue Expression analysis (GTEx Portal) indicates that *LINC01187* is predominantly expressed in kidney tissue (cortex and medulla). TPM: transcripts per million. (B–K) Expression profiles of 9 lncRNAs with real‐time RT‐qPCR analysis in renal biopsies from kidney patients (Pt.) and healthy controls (HC). The lncRNA genes that were examined in human samples have been found deregulated in the kidney of UUO mouse model (conserved lncRNA genes). (J,K) *LINC01187* lncRNA is significantly down‐regulated in individuals with renal disorders compared to HC. The lncRNA levels of each lncRNA were normalized to *GAPDH* and expressed as relative fold of expression to the sample with the minimal fold of expression. *p < 0.05, **p < 0.01, ns: *p* ≥ 0.05 (*t*‐test 2;2)

Informed written consent was obtained from all the participants. Samples were collected following standard procedures from the Research Ethics Committee of each institution. The renal tissues used for the real‐time RT‐qPCR were obtained from the University Hospital of Regensburg (Figure 1J) and the German RWTH Aachen University Hospital (Figure 1K). From the University Hospital of Regensburg, we obtained samples from healthy parts of 4 nephrectomies as healthy controls (HC) and from 5 biopsies of kidney transplant rejections plus 1 biopsy with BK nephropathy as diseased. From the German RWTH Aachen University Hospital, we obtained healthy parts from 4 nephrectomies as HC and 5 biopsies of fibrotic kidneys as diseased. The paraffin‐embedded sections of nephrectomies (renal cell carcinoma), biopsies (DN, RPGN, lupus nephritis‐LN) and healthy parts from nephrectomies (HC), used for the *in situ* experiment, were obtained from archived histopathological material from the Department of Pathological Anatomy, Medical School, Athens, Greece. The sections were 5 μm thick. The demographics for the samples used for the *in situ* are depicted in Table 2.

### 2.2 Animal studies

All procedures regarding animal experimentation were in accordance with the European Union Guidelines for the Care and Use of Laboratory Animals and approved by the local ethics committee of the National Institute for Health and Medical Research (INSERM) (for the Glomerulonephritis and Ischemia/Reperfusion models) and of the Biomedical Research Foundation of the Academy of Athens (BRFAA) (for the UUO model). Animals were housed at constant temperature with free access to water and food.

### 2.3 Unilateral Ureter Obstruction (UUO) mouse model

Adult male C57BL/6 mice at the age of 8 to 12 weeks, were operated in order to ligate the right ureter. During the surgery, the animals were randomly divided in three groups, sham operated (control), 2 days ligated and 8 days ligated, exactly as previously described.^14^, ^26^

### 2.4 Glomerulonephritis model (nephrotoxic serum‐induced, NTS)

The NTS experiments were performed on SV129 male mice aged 8–10 weeks according to a previously described protocol.^14^ Mice received intravenous injections of totally 12 µl NTS/g body weight over 2 consecutive days (days 0 and 1) to induce crescentic GN, while control mice were injected with PBS. Periostin (Postn) expression was inhibited with a cocktail of two different ODNs specifically targeting Postn mRNA, designed using IDT (Integrated DNA Technologies) platform. Scrambled (SCR) non‐specific ODNs were used as control. The ODN sequences were modified with phosphorothioate to prevent their *in vivo* hydrolysis by nucleases (Sigma‐Aldrich). For administration to mice, the ODNs were diluted in normal saline and placed in osmotic mini‐pumps (Alzet, model 1002) which were subsequently implanted subcutaneously in mice, constantly releasing a dose of 0.25 µl per hour which corresponded to a release of 150 pmol/ODN/day. The implantation of mini‐pumps was performed at day 3 after administration of NTS, taking one day for the pump to start functioning. Mice were euthanized 9 days after the first injection (*n* = 4 per group). Renal tissues were collected.

### 2.5 *In vivo* administration

AS and SCR ODN sequences used for *in vivo* administration in mice: AS1 (G*A*G*AGGAACCATCTTCAGCCCTGAGCT*C*C*G); AS1 (G*T*C*TCTCCTGTTTCTCC*A*C*C); SCR1 (C*T*C*TCCGG AGAGCCACCGAGATCTGAG*T*C*A); SCR2 (G*C*T*AT CCTTCCC GCTCT*C*T*T). Residues modified with phosphorothioates to inhibit degradation.

### 2.6 Ischemia/Reperfusion model (I/R)

Eight‐ to 10‐week‐old male mice (*n* = 4 per group) were anesthetized with intraperitoneal injection of ketamine (100 mg/kg)/xylazine (10 mg/kg) and subjected to right kidney nephrectomy in order to enhance the I/R aggression on the remaining kidney. Sham‐operated animals were used as controls. The left renal artery was clamped for 30 min of warm ischemia at 37°C followed by 24 or 72 h of reperfusion. After reperfusion, the mice were euthanized, and renal tissues were collected for subsequent analyses.

### 2.7 Chromatin immunoprecipitation analysis on UUO mouse kidney

Chromatin immunoprecipitation (ChIP) was performed in kidneys of sham‐operated and UUO mice to evaluate the promoters of lncRNA gene as previously described.^14^ The antibodies used were against RNA polymerase II (Millipore), Histone 3 (tri‐methylated K4) (ab8580, Abcam), and a normal rabbit IgG (sc‐ 2027) was used as a control. Precipitated DNAs were examined by real‐time qPCR using the following oligonucleotide primers for the promoter region of *Gm12121* lncRNA: *Gm12121*‐*Prom* (forward, 5′‐GGCCTATTGCTCAGAGAGGA‐3′; reverse, 5′‐GGCCACTTGTGTGTCTGTGT‐3′). All experiments were performed in triplicate. Analysis was carried out via input percent method (ThermoFisher Scientific guidelines). Statistical analysis was performed by Student's *t* test.

### 2.8 RNA extraction and real‐time RT‐qPCR analysis

Total RNA was isolated by using the TRI reagent solution (Sigma) followed by treatment with RQ1 DNase (Promega). RNA concentration was measured by Nanodrop 2000c (ThermoFisher Scientific), and 1 μg of RNA was used for cDNA synthesis using the M‐MLV Reverse Transcriptase (ThermoFisher Scientific) together with random hexamer primers. RNA expression levels of lncRNAs were determined with RT‐qPCR using SYBR Green, and analysis was performed in a LightCycler^®^ 96 Instrument (Roche). Measured values were normalized to GAPDH expression levels using the ΔΔCT method. Statistical analysis was performed by Student's *t* test. For the human samples, the primer sets used were as following: LINC01187‐2 (forward, 5′‐GTCAAGGCAAGCCGTAG AAG‐3′; reverse, 5′‐TGGAGAGTCTGGCTGGAGTT‐3′), RP11‐798M19 (forward, 5′‐ATC GACGCAGAAAATCAAA‐3′; reverse, 5′‐TCAT CCAAAATCCCAAAAGG‐3′), SNHG1 (forward, 5′‐TAACC TGCTTGGCTCAAAGG‐3′; reverse, 5′‐CAGCC TGG AGTGAACACAGA‐3′), MIR17HG (forward, 5′‐GGG TCATTAGG AAAATGCACA‐3′; reverse, 5′‐CAAA CAGCC AGACCAAACTG‐3′), RP5‐1171I10 (forward, 5′‐GGTACG CAGAGGCTGAGAAG‐3′; reverse, 5′‐CCAAGG CTATCCCAA CAAAA‐3′), SNHG5 (forward, 5′‐CACA GTGGAGCAGCTCTGAA‐3′; reverse, 5′‐TCACTGG CTACTCGT CCACA‐3′), SNHG6 (forward, 5′‐CGAAGA GCCGTT AGTCATGC‐3′; reverse, 5′‐CCTC AAAGGCTTT CTTG CAC‐3′), SNHG7 (forward, 5′‐GTGTGTCCC TTGGTGG AGAG‐3′; reverse, 5′‐TCATCTCC AGTGAGCAGACG‐3′), MALAT1 (forward, 5′‐GACGGA GGTTGAG ATGAAGC‐3′; reverse, 5′‐ATTC GGGGCTCTGTAGTCCT‐3′), GAPDH (forward, 5′‐CCTCTGAC TTCAACA GCGAC AC‐3′; reverse, 5′‐AGCCAAA TTCGTTGTCATA CCAG‐3′). To identify the LINC01187 transcript variant expressed in renal biopsies from healthy individuals (Figure S3), we used two different sets of primers: the LINC01187‐2, as mentioned above, which is specific for the 4‐exons transcript, and LINC01187‐1 (forward 5′‐ TGATCAGCACTGGCTTCAAC‐3′; reverse 5′‐AGTCTC AGCA GCAA GGTGGT‐3′), which is specific for the 5‐exons transcript. For the mice samples (NTS, I/R), the primers used were the following: Hprt (forward, 5'‐GGAGCGGTAGCACCTCCT‐3'; reverse 5'‐CTGG TTCATCATCGCTAATCAC‐3'), Gm12121‐Primer 1 (forward, 5′‐ACCC AGGAGCTGCTGATAAA‐3′; reverse 5′‐GCA GGATTGTGGTGAAGGTT‐3′), Gm12121‐Primer 2 (5′‐TCTGGCTGTGCCTAGATGTG‐3′; reverse 5′‐GCTGG ATATCA GGGTGATGG‐3′), Gm12121‐Primer 3 (5′‐TTCCTAGCATG ACCCC TTTG‐3′; reverse 5′‐GCTTGCCC AAAATACA GGAA‐3′).

### 2.9 Microarray analysis of human kidney biopsies

Human kidney biopsy specimens and Affymetrix microarray expression data were obtained within the framework of the European Renal cDNA Bank ‐ Kröner‐Fresenius Biopsy Bank.^27^ Biopsies were obtained from patients after informed consent and with approval of the local ethics committees. Biopsies were processed as previously reported.^28^ Published datasets of glomerular and tubular samples were used in this study (GSE104954, GSE104948). Analysis included datasets from patients with diabetic nephropathy (Glom *n* = 7, Tub *n* = 7), minimal change disease (Glom *n* = 5; Tub *n* = 5), focal segmental glomerulosclerosis (Glom *n* = 10, Tub *n* = 3) and rapidly progressive glomerulonephritis (Glom *n* = 23, Tub *n* = 21). Pre‐transplantation kidney biopsies from living donors (Glom *n* = 18, Tub *n* = 18) were used as control renal tissue. CEL file normalization was performed with the Robust Multichip Average method using RMAExpress (Version 1.0.5) and the human Entrez‐Gene custom CDF annotation from Brain Array version 20 (http://brainarray.mbni.med.umich.edu/Brainarray/Database/CustomCDF/genomic_curated_CDF.asp). To identify differentially expressed genes, the SAM (Significance Analysis of Microarrays) method was applied using SAM function in Multiple Experiment Viewer (TiGR MeV, Version 4.9). A q‐value below 5% was considered to be statistically significant.

### 2.10 RNA probe construction for *in situ* hybridization

Digoxigenin‐labelled RNA probes, sense (control) and anti‐sense to human LINC01187 lncRNA, were prepared to be used for *in situ* hybridization on renal tissue sections. For the preparation of the RNA DIG probe, a region of LINC01187 gene sequence was isolated via PCR using as a template renal cDNA from healthy individuals obtained from the Institute of Pathology, RWTH, Aachen, Germany. T3 and T7 promoter sequences were added to primers when necessary for the function of T3 and T7 polymerases during the *in vitro* transcription for the generation of the probes. Anti‐sense DIG‐RNA probe was made by using T7 polymerase, and sense probe (control) was made by using T3 polymerase. The primers used are the following: a. T3‐LINC01187‐forward, 5′‐GAGGAGAATTAACCCTCACTAAAGGGAGACACCCTGAGGCCAGAAAATA‐3′; LINC01187‐reverse, 5′‐CGCATGATATTCCACTGTGC‐3′, b. T3‐LINC01187‐1‐forward, 5′‐GAGGAGAATTAACCCTCACTAAAGGGAGAAGGGTGCATTTCTTCACCAG‐3′; LINC01187‐1‐reverse, 5′‐TGGAGAGTCTGGCTGGAGTT‐3′, c. LINC01187‐forward, 5′‐CA CCCTGAGGCCAGAAAATA‐3′; T7‐LINC01187‐reverse, 5′‐GAGGAGTAATACGACTCACTATAGGGAGACGCATGATATTCCACTGTGC‐3′, d. LINC01187‐1‐forward, 5′‐AGGGTGCATTTCTTCACCAG‐3′; T7‐LINC01187‐1‐reverse, 5′‐GAGGAGTAATACGACTCACTATAGGGAGACTTCTACGGCTTGCCTTGAC‐3′. The PCR products were analysed through gel electrophoresis using 1% agarose gel in 0.5× Tris/Borate/EDTA (TBE) buffer solution. The products were loaded using 6× blue loading dye (BioLabs), and they were identified using GeneRulerTM 1Κb DNA ladder (Thermoscientific). Τhe gel was transferred to an open UV box, and the desired DNA fragments were cut out with a sterile razor blade and placed in labelled microfuge tubes. The DNA fragments were purified using the NucleoSpin^®^ Gel and PCR Clean‐up kit (MN). DNA concentration and purity were measured by Nanodrop 2000c (Thermo). For the generation of the RNA probes, *in vitro* transcription was performed with 500 ng‐1 μg PCR product using DIG RNA labelling mix (Cat#11277073910 Roche) 2 μl, 10× transcription buffer (Cat#11465384 Roche) 2 μl, Placental ribonuclease inhibitor (Cat#N2111 Promega) 0.5 μl, T3 (Cat#11031171001 Roche) or T7 RNA polymerase polymerase 20 U/μl (Cat#10881775001 Roche) 2 μl and RNase free water to a final volume of 20 μl for 3 h at 37°C. Following the transcription, DNase treatment (Cat#M6101 Promega) and RNA probes clean‐up were carried out using the NucleoFast^®^ 96 PCR kit (MN) according to manufacturers’ instructions.

### 2.11 *In situ* hybridization with Digoxigenin‐RNA probes on paraffin‐embedded sections

The procedure followed has been previously described.^29^ The slides were deparaffinized and rehydrated as following: 2 × 15 min in xylene, 4 min in 100% ethanol, 4 min in 95% ethanol, 4 min in 70% ethanol, 4 min in 50% ethanol, 4 min in distilled water. The sections were washed with 1× PBS, post‐fixed with 4% paraformaldehyde for 20 min, then washed three times with sterile 1× PBS, immersed in a plastic container with 100 mmol/L triethanolamine pH 8 and acetylated by adding dropwise acetic anhydride at a final concentration of 0.25%, while the container was gently agitated for 10 min. The slides were washed with PBS – 0.1% Tween and pre‐hybridized with pre‐warmed hybridization buffer (50% formamide/20× SSC/50× Denhardt's/10 mg/ml yeast RNA, 10 mg/ml salmon sperm DNA) under coverslips for 3 h at 68°C. Then, they were hybridized with 500 ng/ml RNA probe (sense or anti‐sense) diluted in the hybridization buffer in a dry incubator over‐night at 68°C and the next day they were washed twice with pre‐warmed washing buffer (50% formamide/20× SSC/0.1% Tween 20) for 60 min at 68°C. The probe label detection was performed as following: 2 × 15 min washes with B1 (100 mmol/L Tris‐HCl pH 7.5 1 M/150 mmol/L NaCl 4 M/0.1% Tween 20), blocking with B2 (10% inactivated chicken serum in B1) for 3 h, incubation with Anti‐Digoxigenin‐AP (Cat#11093274910 Roche) diluted 1/2000 in B2 overnight at 4°C, 5‐min wash with B1, 2 × 30 min incubation with B3 (100 mmol/L Tris‐HCl pH 9.5 1 M/100 mmol/L NaCl 4 M/50 mmol/L MgCl2 1 M/0.1% Tween 20), incubation with NBT/BCIP (Cat#11681451001 Roche) in B3 (20 μl/ml), 3 × 10 min washes with 1× PBS, post‐fixation with 4% paraformaldehyde and washes with 1× PBS and distilled water. The sections were mounted in Kaiser's gelatin (MERCK 1092420100) and viewed under the optical microscope.

### 2.12 Cell Culture and Overexpression studies in HEK293 cell line

HEK293 cells were cultured in Dulbecco's modified Eagle's medium (DMEM) with high concentration of glucose (4.5 g/L) (Biowest, Cat. No L0106‐500), supplemented with 10% foetal bovine serum (FBS), 2 mmol/L glutamine and penicillin/streptomycin. Transient transfections in HEK293 cells were performed using the calcium phosphate method, as previously described.^30^ Plasmids used for transfections were pcDNA3.1‐LINC01187 constructed with gene synthesis by GenScript Biotech, pcDNA3.1‐GFP and empty pcDNA3.1 as the control construct. pcDNA3.1‐LINC01187 or empty pcDNA3.1 was co‐transfected with pcDNA3.1‐GFP, with a ratio 3:1, and transfected cells were detected by GFP expression.

### 2.13 Proliferation and Apoptosis detection assays

We evaluated the role of LINC01187 in cell proliferation and apoptosis in HEK293 cells overexpressing LINC01187. Therefore, we determined the basal levels of Ki‐67 for the proliferation and of Annexin V/7‐AAD for the apoptosis without the administration of a toxic, proapoptotic stimulus. More specifically, for the proliferation detection assay, cells were stained with PerCP/Cyanine5.5 anti‐human Ki‐67 antibody (Cat#350520 Biolegend) (1:50) using the eBioscience™ Foxp3/Transcription Factor Staining Buffer Set (Cat#00‐5523‐00). For the apoptosis detection assay, cells were stained with PE Annexin V Apoptosis Detection Kit with 7‐AAD (Cat#640934 Biolegend). The flow cytometer used was FACS‐ARIA‐III (Becton Dickinson Biosciences). GFP^+^ gated cells (transfected) were analysed for the emission of Ki‐67 or Annexin V/7‐AAD signal. Analysis was performed using FlowJoTM V10.

## 3 RESULTS

### 3.1 Identification of conserved human lncRNAs with potential involvement in renal diseases

Using genome‐wide transcriptomic analysis we have previously identified several mouse lncRNAs differentially expressed in the kidney of UUO model.^14^ These data raised the question of whether our newly identified lncRNAs are conserved and functionally important in humans.

To tackle this question, we initially compared the gene sequences of mouse lncRNAs with human homologues and other vertebrate species (Figure S1). We also compared the promoter sequences of these genes, considering the significance of sequence conservation at the promoter level equally important. This notion is a commonly emerging paradigm in lncRNA analysis, where lncRNA genes found in syntenic genomic regions between species exhibit low conservation in the RNA sequence, yet they retain high conservation in the promoter sequence.^23^, ^24^, ^25^ This conservation is indicative of shared functional importance. Therefore, we were able to identify 18 conserved lncRNAs, 11 of which were up‐regulated [*AI504432*, *Gm13889*, *A430104N18Rik (Μir142hg)*, *Gm20645*, *Snhg5*, *Neat1*, *Snhg1*, *Snhg6*, *Mir17hg*, *Malat1* and *Snhg7*] and 7 were down‐regulated [*500016L03Rik* (*Lhx1os*), *Gm17750*, *1700022N22Rik*, *Fam120aos*, *9130409J20Rik*, *2500002B13Rik*, *Gm12121*] in the kidney of mouse UUO model (Table 1). We excluded *AI504432* (human *KCNA3*), *Gm13889* (human *C11ORF96*) and *Gm20645* (human *RP11*‐*373L24*.*1*) from further analysis because it is highly likely for the human homologues to either express a protein product or being part of a protein‐coding gene. In most cases, promoter sequences of the identified lncRNAs are conserved into the evolutionary clade of placental mammals (Figure S1A–D and F–O), suggesting a conserved expression pattern.

**TABLE 1:** Identification of human homologues of UUO‐affected mouse lncRNAs with statistically significant differences when comparing sham‐operated (SO) with 8‐days post ligation (8D) or SO with 2‐days post ligation (2D) animals

Mouse lncRNA genes	Fold change	*p*‐Value	Corresponding human lncRNA	Conservation into RNA	Conservation into Promoter
Up‐regulated lncRNAs					
AI504432	8.275482	5E‐07	KCNA3 (NR_109846.1 or NR_109845.1)	YES	YES
Gm13889	6.822517	2E‐12	C11ORF96	YES	YES
A430104N18Rik (Μir142hg)	6.6707441	0.0001	RP5‐1171I10.5 (MIR142 long version)	YES	YES
Gm20645	2.9815745	0.0082	RP11‐373L24.1	YES	NO
Snhg5	2.8943278	0.0017	SNHG5	NO	YES
Neat1	2.7959217	5E‐05	NEAT1	YES	YES
Snhg1	2.6327966	0.0006	SNHG1	YES	YES
Snhg6	2.1701416	0.0083	SNHG6	YES	NO
Mir17hg	2.161294	0.0154	MIR17HG	YES	YES
Malat1	2.059809	0.0041	MALAT1	YES	YES
Snhg7	1.9909213	0.0276	SNHG7	NO	YES
Down‐regulated lncRNAs					
1500016L03Rik (Lhx1os)	0.3879853	0.0024	LOC102723471	NO	YES
Gm17750	0.3809115	0.0039	TMEM161B‐AS1 (NR_105020.1)	YES	YES
1700022N22Rik	0.3320594	0.0143	LINC00235	YES	YES
Fam120aos	0.3012063	0.0001	Fam120aos	YES	YES
9130409J20Rik	0.2364907	0.0006	LINC00675	YES	YES
2500002B13Rik	0.2036775	3E‐06	RP11‐798M19.6	NO	YES
Gm12121	0.1597814	9E‐08	LINC01187	YES	YES

Next, we chose 9 lncRNAs for further investigation, based on whether human homologues are expressed in renal tissue (even not exclusively). In particular, we examined the tissue‐specific gene expression pattern by using the Human Genotype‐Tissue expression database (GTEX) (Figures 1A and S2). Interestingly, only in the case of *LINC01187*, the kidney is the predominant tissue of expression (Figure 1A), while all other lncRNAs are expressed in a more ubiquitous manner (Figure S2). To verify the expression in the renal tissue and test whether these lncRNAs are differentially expressed between healthy and diseased individuals, we performed real‐time RT‐qPCR assays in renal biopsies from kidney transplants rejections and BK nephropathy compared to healthy kidneys. Although most lncRNAs were detected in renal tissue (Figure 1B–I), in agreement with our bioinformatic analysis (Figure S2), only *LINC01187* is differentially expressed between healthy and pathological samples (Figure 1J). Two alternative transcripts for *LINC01187* have been reported in NCBI RefSeq annotation and Ensembl/GENCODE annotation. Using exon‐specific primers for real‐time RT‐qPCR assays, we found that healthy subjects express the Ensembl/GENCODE annotated *LINC01187* transcript variant with four exons (Figure S3). The differential expression of *LINC01187* was further experimentally validated in an independent cohort of kidney samples from patients with renal fibrosis (Figure 1K). These data raise the intriguing hypothesis that this conserved lncRNA (*Gm12121* in mouse or *LINC01187* in human) may be involved in kidney diseases.

### 3.2 *LncRNA* *Gm12121* is down‐regulated in animal models of renal diseases

**FIGURE 2:**
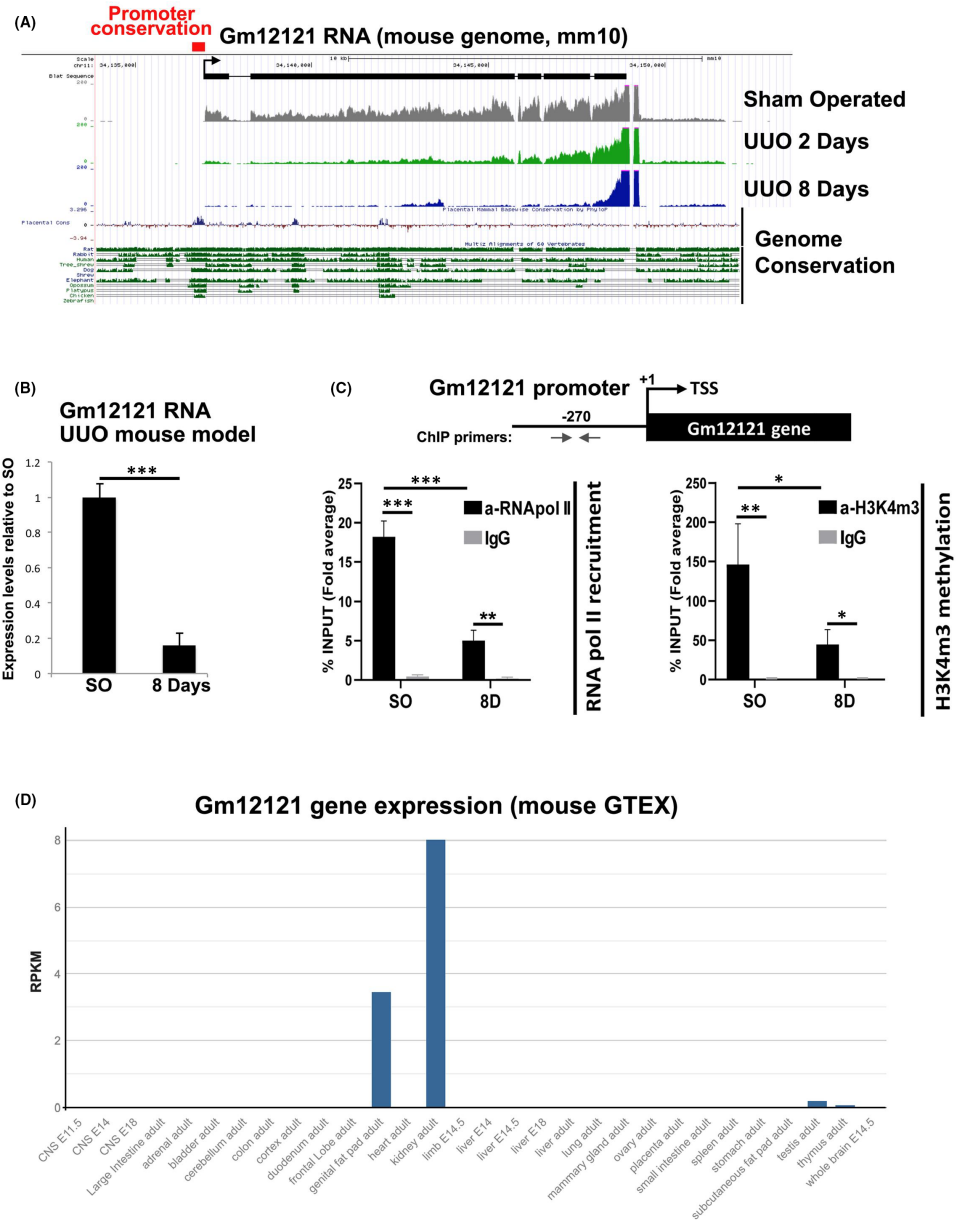
Analysis of *Gm12121* lncRNA at both RNA and chromatin organization levels. (A) Visualization of RNA‐seq peaks for *Gm12121* lncRNA using the UCSC Genome Browser in representative sham‐operated (SO), 2 days ligated (2D) and 8 days ligated (8D) UUO renal samples. Conservation analyses indicate that both promoter and RNA sequences of *Gm12121* lncRNA are conserved among many species including mice and humans. (B) Expression analysis of *Gm12121* in UUO mouse model. (C) Chromatin immunoprecipitation experiments with antibodies against RNA polymerase II (RNA pol) and H3K4m3 (as indicated) in conjunction with real‐time RT‐qPCR analysis. Schematic representation of the relative genomic position of the primers used for RT‐qPCRs experiments is indicated at top panel. For these experiments, chromatin samples were prepared from kidneys of SO *n* = 3 and 8D mice *n* = 3, *p < 0.05, **p < 0.01, ***p < 0.001 (*t*‐test 2;2). (D) Mouse Genotype‐Tissue Expression analysis (GTEx Portal) shows that *Gm12121* lncRNA is predominantly expressed in adult kidney

In agreement with the human data, *Gm12121*, the mouse homologue of *LINC01187*, is dramatically down‐regulated in the kidney of UUO mouse model, 2 (2D) and 8 days (8D) post‐ligation as compared to sham‐operated (SO) animals (Figure 2A,B). Chromatin immunoprecipitation assays combined with real‐time qPCR analysis for the *Gm12121* promoter region demonstrated a significant decrease in the binding of RNA polymerase II and tri‐methylation of Lysine 4 of Histone 3 (Figure 2C). These data indicate that the promoter of *Gm12121* in UUO is repressed at the chromatin activation level, which leads to transcriptional inactivation. The promoter region of mouse *Gm12121* exhibits remarkable conservation among humans and many other vertebrates and mammalian species (Figure S4), suggesting a conserved expression pattern. *Gm12121* is also predominantly expressed in the kidney (Figure 2D), similar to its human homologue (Figure 1A).

**FIGURE 3:**
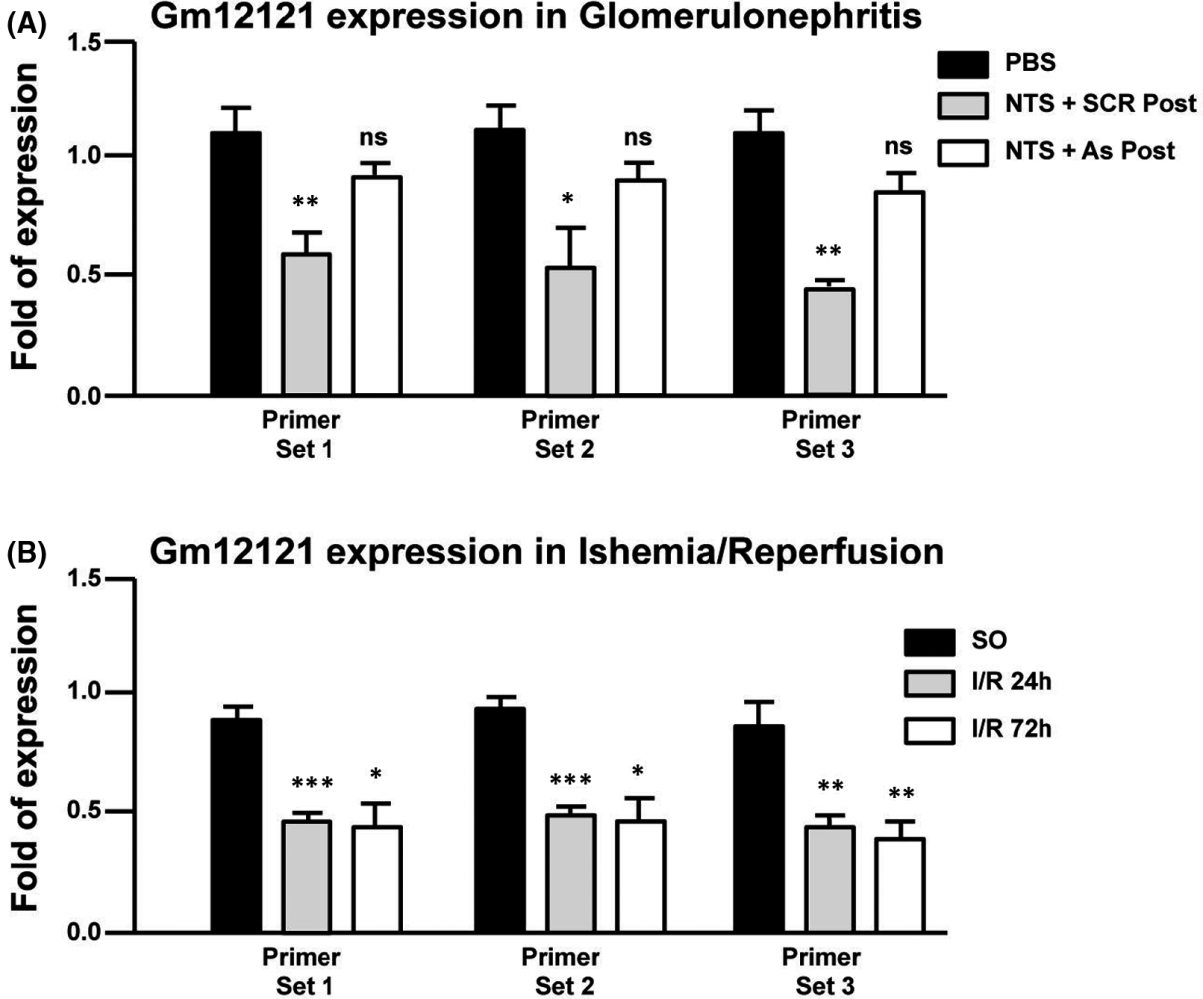
Changes in the expression levels of *Gm12121* lncRNA gene in NTS‐induced Glomerulonephritis and I/R animal models of kidney diseases. Real‐time RT‐qPCR analysis of the expression profile of *Gm12121* in the: (A) model of glomerulonephritis, induced by intravenous administration of nephrotoxic serum (NTS). Treatment with anti‐sense (AS) oligonucleotides against periostin is protecting kidneys from the development of CKD^31^ and is sufficient to restore *Gm12121* expression levels. PBS: animals injected with PBS; NTS + SCR Post: animals injected with NTS and treated with scrambled oligonucleotides; NTS + As Post: animals injected with NTS and treated with anti‐sense oligonucleotides against periostin. (B) Ischemia/reperfusion (I/R) model of acute kidney injury. SO: sham operated; I/R 24 and 72 h: tissues are dissected 24 or 72 h after ischemia/reperfusion, respectively. Primer sets 1, 2, 3 are described in Materials and Methods section. The RNA levels of *Gm12121* were normalized to *Hprt*. *p < 0.05; **p < 0.01, ***p < 0.005, ns: *p* ≥ 0.05 (*t*‐test 2;2), *n* = 4 animals per group of treatment

To explore the possibility that alterations in the expression of *Gm12121* might also occur in pathological conditions other than the ureteric obstruction, we investigated two other models of renal diseases: the NTS‐induced glomerulonephritis, a severe model of CKD and the I/R, a model of acute kidney injury (AKI), in which a maladaptive repair response is associated with secondary chronic kidney disease development. *Gm12121* expression is significantly down‐regulated in NTS‐induced glomerulonephritis and I/R at 24 and 72 h post‐injury (Figure 3). Administration of anti‐sense (AS) oligonucleotides against periostin, which protects kidneys from the development of CKD,^31^ is sufficient to restore *Gm12121* expression levels to their normal values (Figure 3A). Collectively, these findings demonstrate the association between expression levels of *Gm12121* and renal diseases.

### 3.3 *LINC01187* is down‐regulated in diabetic nephropathy and rapidly progressive glomerulonephritis in humans

**FIGURE 4:**
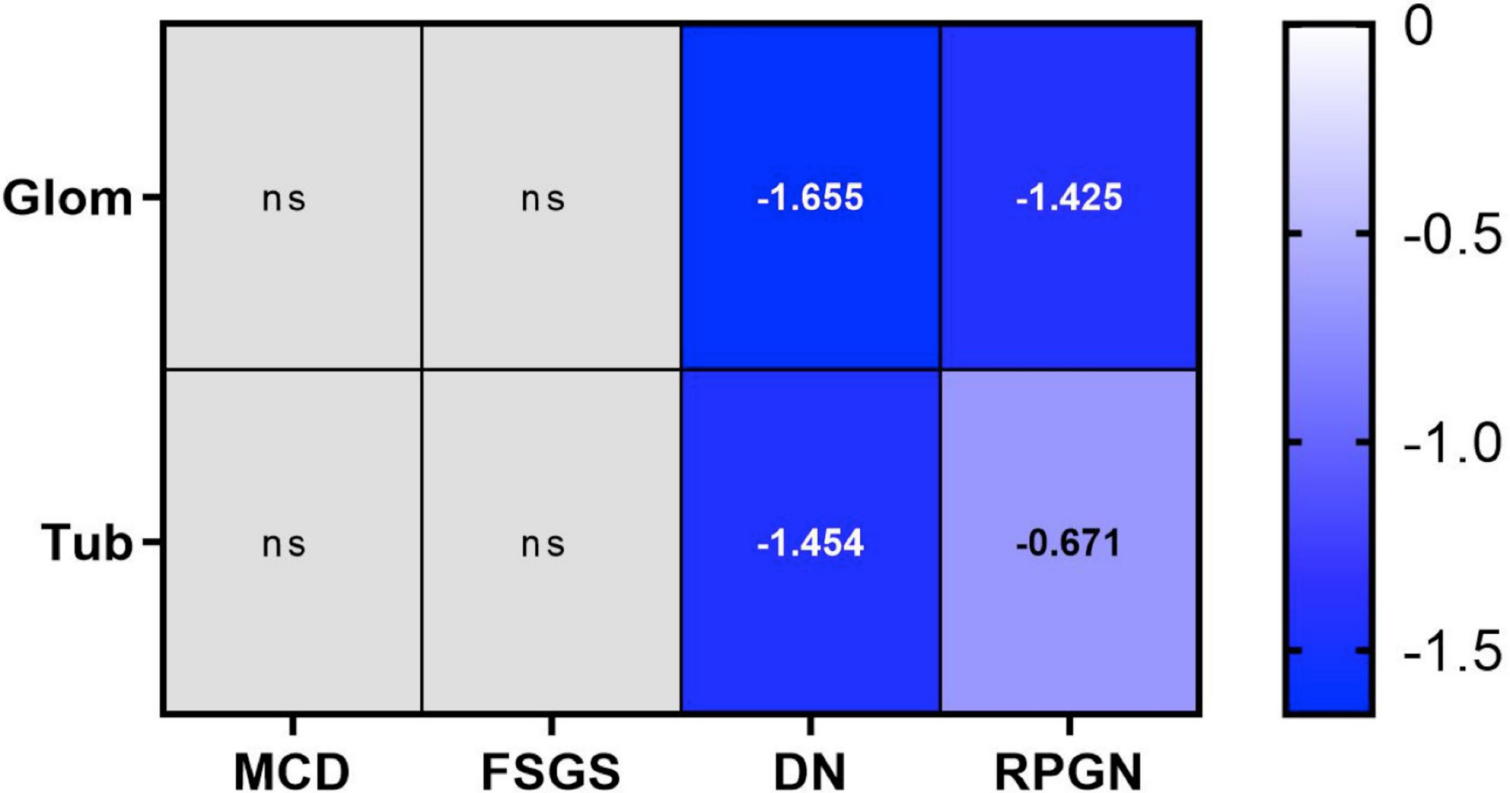
Gene expression analysis of *LINC01187* gene in the glomerular and tubulointerstitial compartment of manually micro‐dissected kidney biopsies from patients with different kidney diseases. Values are expressed as log2‐fold change compared to controls (living donors, LD). A *q*‐value below 5% was considered to be statistically significant. ns: *p* ≥ 0.05. MCD, minimal change disease; FSGS, focal segmental glomerulosclerosis; DN, diabetic nephropathy; RPGN, rapidly progressive glomerulonephritis

To further examine the correlation between the gene expression of this conserved lncRNA and human kidney diseases, we assessed the RNA expression of *LINC01187* in the glomerular and tubulointerstitial compartments from patients with DN, focal segmental glomerulosclerosis (FSGS), minimal change disease (MCD) and RPGN in comparison with living donor (LD). We found that *LINC01187* is significantly reduced in two specific renal diseases, DN and RPGN, but not in MCD or FSGS. The down‐regulation in each disease is observed in both tubular and glomerular compartments (Figure 4).

**FIGURE 5:**
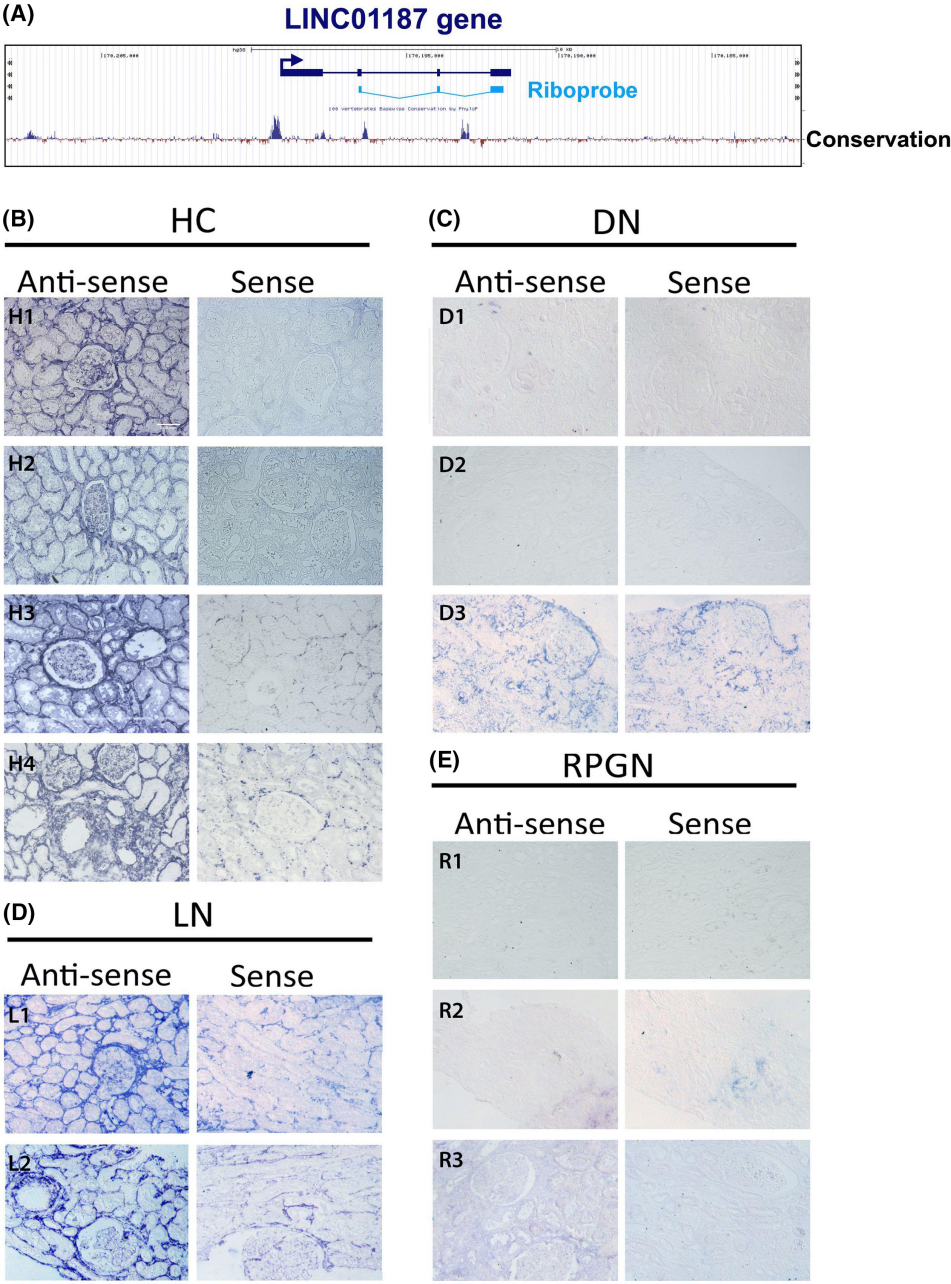
*LINC01187* lncRNA expression is down‐regulated in renal tissue from patients with diabetic nephropathy and rapidly progressive glomerulonephritis. (A) Schematic representation of the relative position of *LINC01187* lncRNA (dark blue) in the genome and the riboprobe (light blue) used for the RNA *in situ* hybridization analysis. (B‐E) RNA *in situ* hybridization analysis with anti‐sense (experimental) and sense probes (internal control) of *LINC01187* on renal tissues from nephrectomies and biopsies. Data from healthy renal tissues from nephrectomies, due to cancer, from four individuals (H1, H2, H3, H4) are presented in (B). Data from biopsies of diabetic nephropathy (DN) from 3 individuals (D1, D2, D3) are presented in (C). Data from biopsies of lupus nephritis (LN) from 2 individuals (L1, L2) are presented in (D). Data from biopsies of rapidly progressive glomerulonephritis (RPGN) from 3 individuals (R1, R2, R3) are presented in (E). Representative scale bar 0.08 mm on first picture

Using *in situ* hybridization with a DIG‐labelled, anti‐sense riboprobe specific for *LINC01187* (Figure 5A), we tested whether down‐regulation of this lncRNA can be also detected with a morphological assay in the same pathological conditions (DN and RPGN) compared to healthy regions of kidney nephrectomies from cancer patients (Table 2). In agreement with the results from gene expression analysis, a strong reduction of *LINC01187* is observed in DN and RPGN renal biopsies, while this reduction is not noticeable in biopsies from lupus nephritis (LN) patients (Figure 5B–E). As an internal, negative control, we have also used a sense riboprobe not able to be hybridized with *LINC01187* RNA. The *LINC01187*‐specific signal was localized around glomeruli and the tubules, suggesting a predominant interstitial distribution.

**TABLE 2:** Demographics and clinical characteristics of human samples used for *in situ* hybridization experiments

ID	Age at Bx*	Sex	Ethnicity	eGFR***	Comorbidities**	Proteinuria	Glomerular hematuria	Preexisting CKD	RPGN	Diagnosis
18638	42	M	Caucasian	92	−	+	+	−	−	LN
14637	53	F	Caucasian	84	−	+	+	−	−	LN
11422	69	F	Caucasian	22	+	−	+	−	+	AAV
17348	73	F	Caucasian	20	+	+	+	−	+	AAV
587	65	M	Caucasian	21	−	+	+	−	+	AAV
8567	80	M	Caucasian	33	+	+	−	+	−	DN
4136	44	M	Caucasian	9	+	+	−	+	−	DN
14051	59	F	Caucasian	20	+	+	−	+	−	DN
13448	62	F	Caucasian	N/A	N/A	−	−	N/A	−	Healthy****
15360	77	F	Caucasian	N/A	N/A	−	−	N/A	−	Healthy****
16229	41	F	Caucasian	N/A	N/A	−	−	N/A	−	Healthy****
16780	47	F	Caucasian	N/A	N/A	−	−	N/A	−	Healthy****

Note

Healthy subjects (*n *= 4), subjects with lupus nephritis (*n *= 2), with ANCA‐associated vasculitis *n *= 3, with diabetic nephropathy (*n *= 3).

Abbreviations: AAV, ANCA‐associated vasculitis; Bx, Biopsy; CKD, chronic kidney disease; DN, diabetic nephropathy; F, female; LN, lupus nephritis; M, male; N/A, not applicable; RPGN, Rapidly progressive glomerulonephritis.

* Or nephrectomy

** Hypertension (HTN), dyslipidemia, diabetes mellitus (DM)

*** According to CKD‐EPI

**** Healthy part of nephrectomies.

In addition, we correlated the *LINC01187* expression levels from *in situ* hybridization (shown in Figure 5b) with the corresponding eGFR rates (shown in Table 2). This correlation analysis indicates that expression levels of *LINC01187* are positively correlated with eGFR rates (Figure S5), suggesting that its expression is related to an adequate kidney function.

### 3.4 *LINC01187* overexpression in human kidney cells protects from apoptosis

**FIGURE 6:**
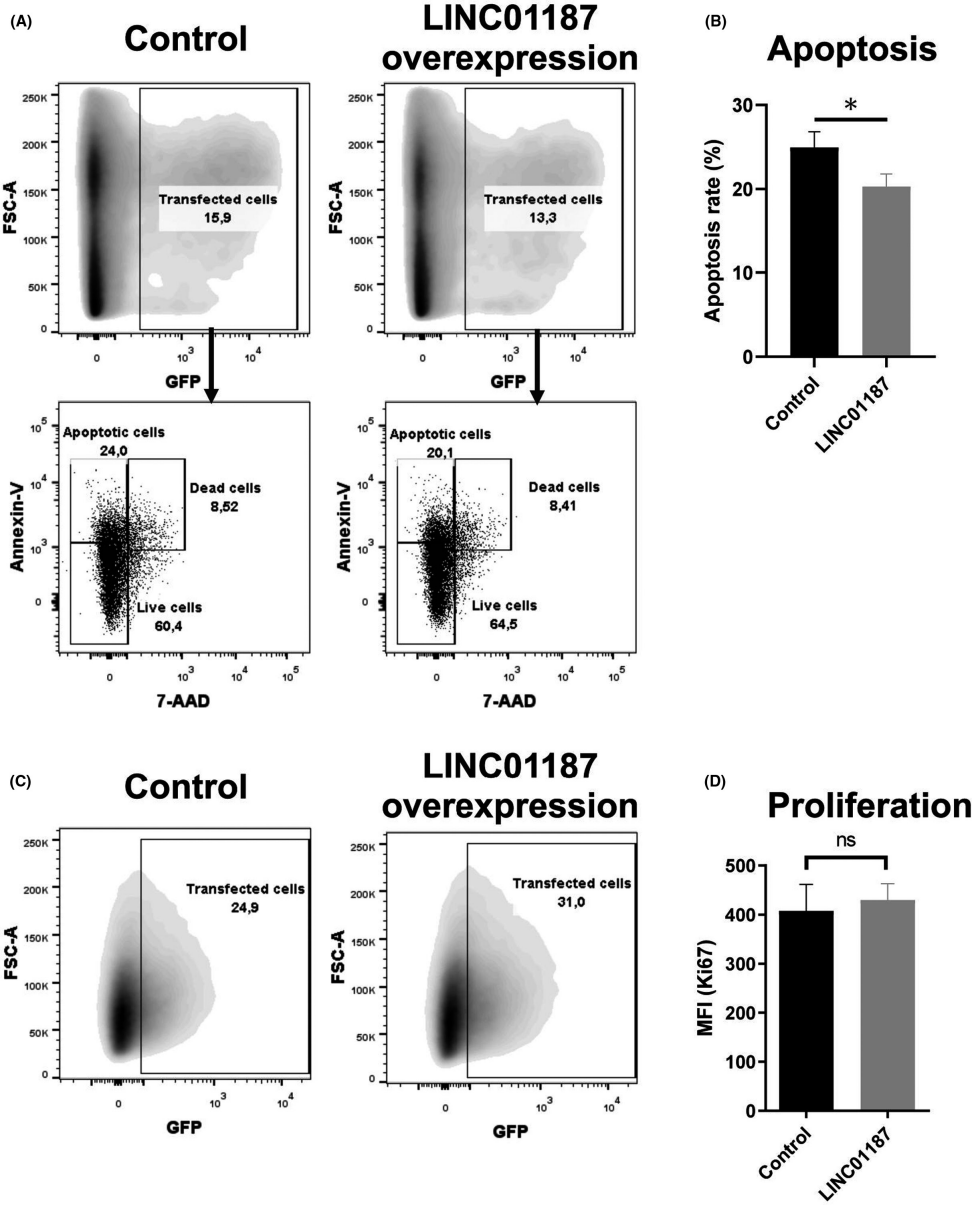
*LINC01187* overexpression in renal cells reduces apoptotic rate without affecting proliferation. (A) Representative flow cytometry gating analysis of transfected cells (GFP^+^) in control condition (co‐transfection of GFP plasmid with empty pcDNA3.1 vector) and *LINC01187* overexpression condition (co‐transfection of GFP plasmid with pcDNA3.1 containing *LINC01187* sequence insert) for Annexin V and 7‐AAD markers. (B) Apoptotic rate in control condition and *LINC01187* overexpression condition. Three biological replicates per condition; t‐test; *p < 0.05. (C) Representative flow cytometry gating analysis of transfected cells (GFP^+^) in control condition (co‐transfection of GFP plasmid with empty pcDNA3.1 vector) and *LINC01187* overexpression condition (co‐transfection of GFP plasmid with pcDNA3.1 containing *LINC01187* sequence insert). (D) Mean fluorescent intensity (MFI) of Ki‐76 in control condition and *LINC01187* overexpression condition. Three biological replicates per condition; *t*‐test; *p* = 0.58 (*ns*)

*LINC01187* down‐regulation in renal diseases, such as DN and RPGN, suggests a potential involvement of this RNA in disease pathophysiology. To investigate this hypothesis, we initially performed gain‐of‐function experiments in a cell line that does not express *LINC01187*. To this end, we conducted overexpression experiments in HEK293 kidney cells. *LINC01187* overexpression protects HEK293 cells from apoptosis, quantified by a FACS‐based Annexin V/7‐AAD positive/negative staining (Figure 6A,B). On the other hand, under the same experimental conditions proliferation rate is not affected as measured by a FACS‐based Ki‐67 assay (Figure 6C,D).

Together, these observations imply a negative association of *LINC01187* with kidney diseases through a potential anti‐apoptotic action.

## 4 DISCUSSION

Advances in genomics have uncovered a large number of lncRNAs involved in human pathologies, including renal diseases. Gene regulatory mechanisms involving both coding and non‐coding RNAs are critical to renal function as well as disease progression.^32^, ^33^ Yet, the functions of lncRNAs in renal pathophysiology remain poorly understood.

We have previously identified several lncRNAs associated with renal dysfunction in mouse disease models using genome‐wide approaches.^14^ Here, to further address the significance of these findings in the context of human kidney diseases, we first screened these murine lncRNAs, which are differentially expressed in the kidney of UUO model, for conservation in the human transcriptome and then focused on their regulation in kidney diseases. We uncovered a negative association between *LINC01187* expression and specific types of kidney disorders. The differential expression of this new molecular player was firmly confirmed and established at the RNA and chromatin organization levels. Interestingly, we documented that *LINC01187* is significantly down‐regulated in human renal pathologies, including DN and RPGN, as well as additional murine models of renal pathologies, including NTS‐induced glomerulonephritis and I/R. Moreover, we were able to show its *in situ* expression pattern around glomeruli and tubules in healthy conditions compared to other kidney diseases, suggesting association of *LINC01187* reduction with specific renal pathologies. In this respect, it will be interesting in future studies to assign its expression to one or more specific renal cell types.

Another critical question arising from our data is about the underlying molecular mechanism that determines in which renal pathologies the expression of *LINC01187* is reduced. We propose that *LINC01187* is more likely to be down‐regulated in pathologies where the glomeruli capillaries and eGFR are mostly affected (e.g. DN and RPGN). Conversely, *LINC01187* is expressed normally in the conditions where the kidneys do not immediately collapse, there are only diffuse glomeruli defects and the eGFR usually falls within the normal range (e.g. LN, MCD, FSGS), suggesting a potential protective role. Therefore, we hypothesize that *LINC01187* is a downstream target gene of a renoprotective signalling pathway that upon cellular toxic stimuli in renal tissue is inactivated and inhibits this gene. In agreement with this hypothesis, we present novel data suggesting that *LINC01187* overexpression in human kidney cells inhibits cell death. These data imply an *LINC01187*‐mediated anti‐apoptotic function. Thus, we propose a negative association of *LINC01187* expression with kidney diseases that may suggest a protective role. These observations add a new layer of molecular complexity in understanding the involvement of lncRNAs in kidney diseases.

Along these lines, few other lncRNA genes have also been associated with impaired kidney function. It has been shown that *MALAT1* may serve as a biomarker of renal ischemia/reperfusion, a major cause of AKI. In particular, plasma and kidney biopsies of patients with AKI have increased levels of *MALAT1* lncRNA.^34^ Moreover, Yang et al. revealed that *NEAT1* lncRNA mediates renal tubulointerstitial fibrosis and epithelial‐mesenchymal transition (EMT). Inhibition of *NEAT1* in a diabetic kidney disease mouse model had a protective effect against EMT and renal fibrosis.^35^ These pioneering studies provide a proof of concept paradigm suggesting the importance of long non‐coding transcriptome in kidney function.

Aberrant expression of lncRNAs has been associated with diverse pathologies including cancer, cardiovascular, neurological and metabolic diseases. Despite the wealth of information on this issue, only during the last decade we began to understand the molecular mechanisms through which lncRNAs regulate cellular function.^16^, ^36^, ^37^ There is increasing evidence that lncRNAs are involved in gene regulation and expression in various ways, acting as scaffolds, decoys, guides, enhancers and through cis or trans genomic targeting, dependent on their position with respect to neighbouring protein‐coding genes.^38^, ^39^ Along these lines, it is of interest to note that *LINC01187* gene is located in close genomic proximity with *FOXI1* and *LCP2* protein‐coding genes. The genomic distance between *FOXI1* and *LINC01187* gene is 81.8 kb, whereas between *LCP2* and *LINC01187* is 47 kb. Both protein‐coding genes are playing important roles in kidney function. Specifically, *FOXI1* is essential for kidney homeostasis by modulating the expression of *SLC4A1*/*AE1*, *SLC4A9*/*AE4*, *ATP6V1B1* and the differentiation of intercalated cells in the epithelium of distal renal tubules. Mutations in these genes cause defective kidney acid secretion leading to distal renal tubular acidosis.^40^, ^41^, ^42^ Interestingly, both *FOXI1* and *LINC01187* genes were recently reported to be involved in chromophobe renal cell carcinoma.^43^ On the other hand, high expression levels of *LCP2* gene have been associated with human clear cell renal cell carcinoma (ccRCC).^44^ These observations may indicate the involvement of *LCP2* in the proliferation and survival properties of renal cells. Thus, it would be extremely interesting to investigate in future studies the potential co‐regulatory mechanisms and inter‐play between these coding genes and *LINC01187* non‐coding gene. Besides, studies that will utilize knockout mice technology may further uncover the protective function of *LINC01187* in renal tissue.

Our results from *in situ* hybridization studies suggested that a specific cell type of the renal interstitium may be responsible for the expression of *LINC01187* in normal tissue. Our attempts to identify this cell type by culturing existing renal interstitial cell lines were not successful. This is not surprising, since several distinct cell types may be harboured in the renal interstitium, as recent studies suggest.^45^

In conclusion, these data provide evidence that *LINC01187* down‐regulation is involved in renal pathogenesis, whereas its expression may imply a protective role. The correlation between *LINC01187* expression pattern and renal diseases provides novel insight into the mechanisms by which lncRNAs mediate renal pathogenesis. Finally, this is the first study, to our knowledge, that suggests a protective role for the newly discovered *LINC01187* lncRNA in any tissue or organ.

## CONFLICTS OF INTEREST

The authors confirm that there are no conflicts of interest.

## AUTHOR CONTRIBUTION

TM performed experiments, analysed the data and wrote the manuscript. VK, NP, PK, SRW, HG, MB, BB, MTL, CDC, PB, SD, DTB and CC performed experiments and prepared and provided biological samples. AC and PKP coordinated and directed the entire project and wrote the manuscript. All authors read and approved the final manuscript.

## ETHICAL APPROVAL

Human samples were collected after signed consent forms were provided, following standard procedures from the Research Ethics Committee of each Institution. All procedures regarding animal experimentation were in accordance with the European Union Guidelines for the Care and Use of Laboratory Animals and approved by the local ethics committee of the National Institute for Health and Medical Research (INSERM) (for the NTS‐induced glomerulonephritis and I/R models) and of the Biomedical Research Foundation of the Academy of Athens (BRFAA) (for the UUO model).

## Supporting information

Figures S1‐S5

Figure Legends

## Data Availability

The data that support the findings of this study are available from the corresponding authors, Aristidis Charonis and Panagiotis K Politis, upon request. Published datasets were also used in this study (GSE104954, GSE104948).
