# Supplementary material for: Down‐regulation of human long non‐coding RNA LINC01187 is associated with nephropathies
Source: J Cell Mol Med. 2023 Apr 13;27(9):1192–205. doi: 10.1111/jcmm.17014 (PMC10148052; doi:10.1111/jcmm.17014)
Supplement: Supplementary file 2 — Figure Legends [file JCMM-27-1192-s002.docx]

**Supplementary Figure Legends**

**Supplementary Figure S1. Conservation of selected mouse lncRNA gene loci among various vertebrate species using UCSC Genome Browser.**

These mouse lncRNAs have been found differentially expressed in the kidney of Unilateral Ureteric Obstruction (UUO) model (14).

**Supplementary Figure S2. Human Genotype-Tissue Expression analysis (GTEx Portal) for the selected lncRNAs indicates their expression in various tissues including kidney.**

**Supplementary Figure S3. The 4-exon variant of *LINC01187* is the predominant version that is expressed in the human kidney.**

**(A)** Schematic representation of the two variants of *LINC01187* transcript. In particular, based on the NCBI RefSeq annotation, *LINC01187* consists of five exons (upper panel) while the Ensembl/GENCODE annotation refers to four exons (lower panel). The primer sets that were used to detect the two alternative variants of *LINC01187* are also indicated. **(B)** To determine the relative expression of the two *LINC01187* variants, we analyzed renal biopsies of healthy individuals by performing absolute quantification by RT-qPCR. Using exon-specific primers, our data revealed that healthy subjects almost exclusively express the Ensembl/GENCODE annotated *LINC01187* transcript variant with four exons.

**Supplementary Figure S4. Visualization of *Gm12121* lncRNA genomic loci using UCSC Genome Browser.**

The promoter region exhibits high conservation among human and many other vertebrate and mammalian species, as indicated.

**Supplementary Figure S5. Kidney function in association with *LINC01187* expression levels.** For the calculation of *LINC01187* levels, the ratio of color segmentation between anti-sense *LINC01187* probe and sense *LINC01187* probe (negative control) was assessed per sample following ISH image analysis. eGFR was used to indicate kidney function. Correlation coefficient is derived from Pearson’s test. eGFR: estimated glomerular filtration rate, A.U.: arbitrary unit, DN: diabetic nephropathy, RPGN: rapidly progressive glomerulonephritis, HC: healthy control, LN: lupus nephritis.
