# Supplementary material for: Down‐regulation of human long non‐coding RNA LINC01187 is associated with nephropathies
Source: J Cell Mol Med. 2023 Apr 13;27(9):1192–205. doi: 10.1111/jcmm.17014 (PMC10148052; doi:10.1111/jcmm.17014)

Supplementary Figure S1

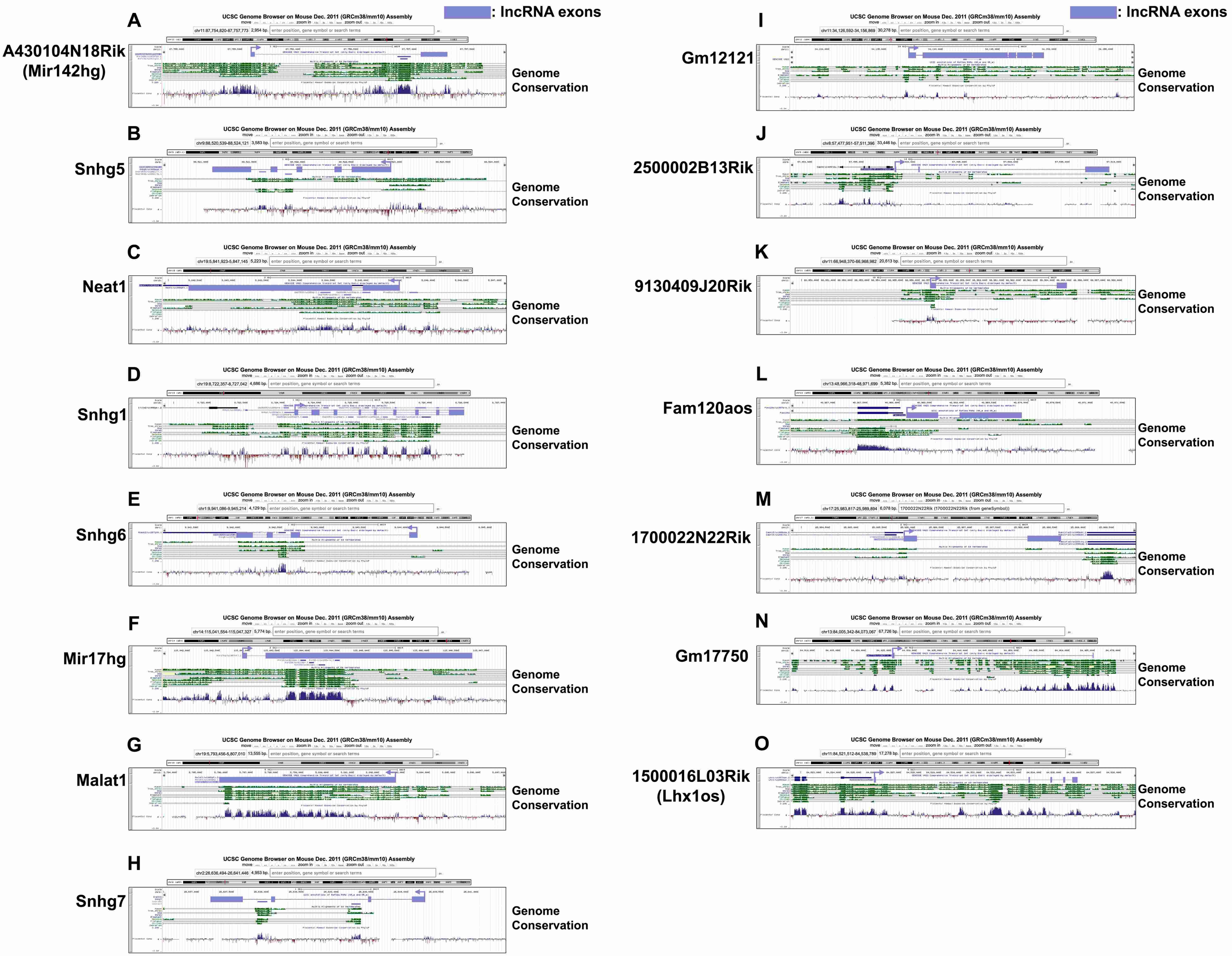

Supplementary Figure S2

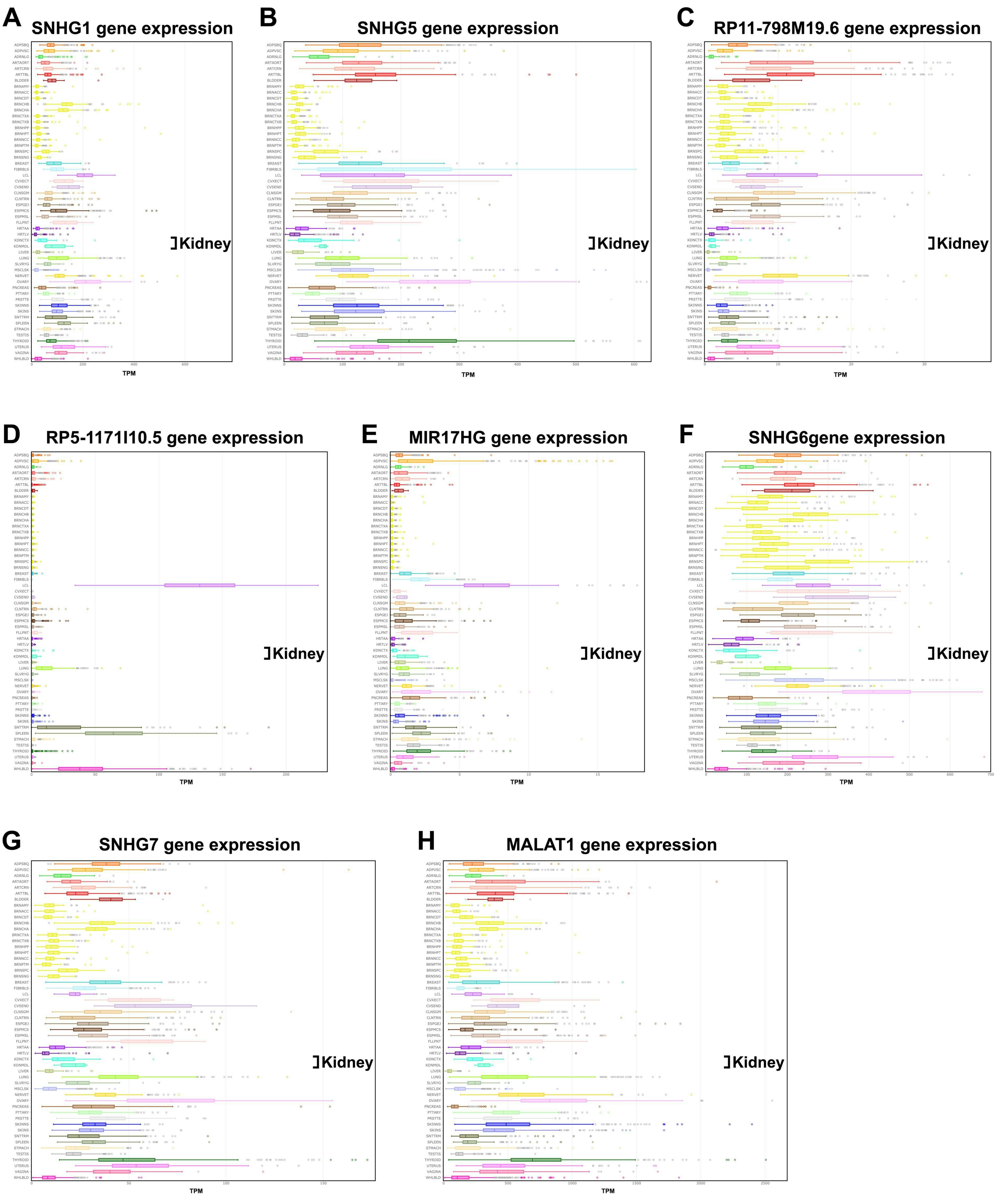

Supplementry Figure S3

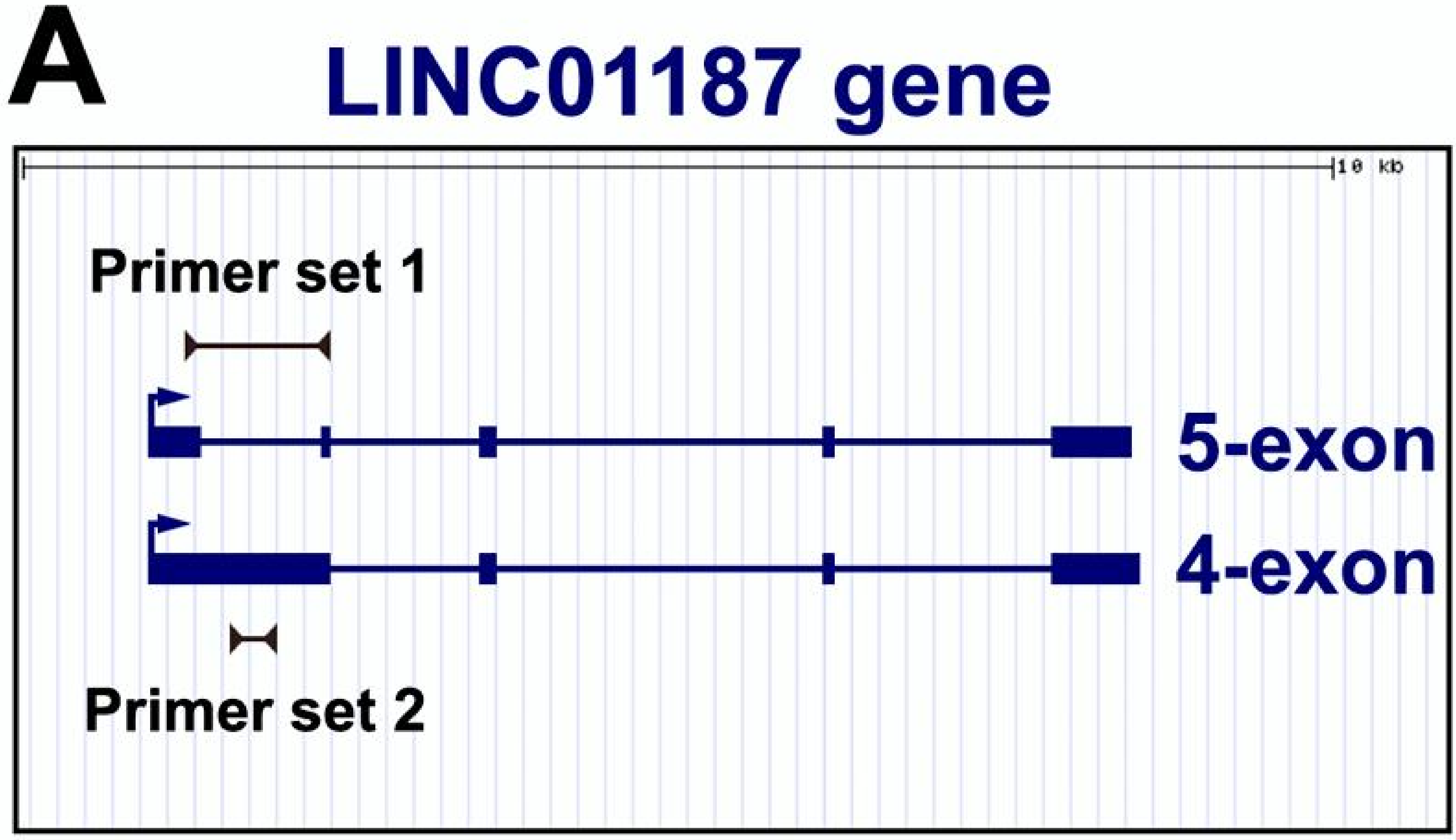

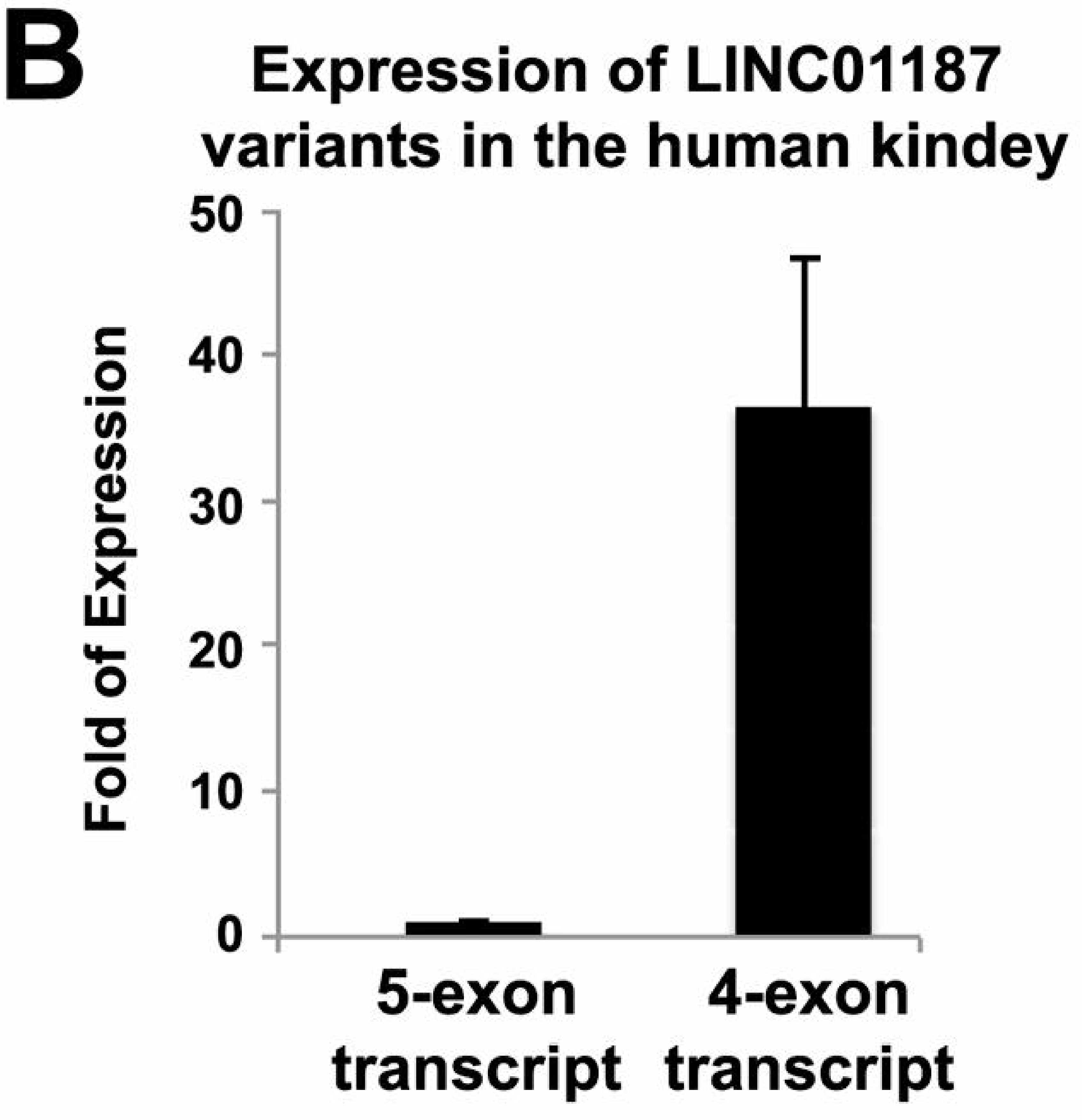

Supplementary Figure S4

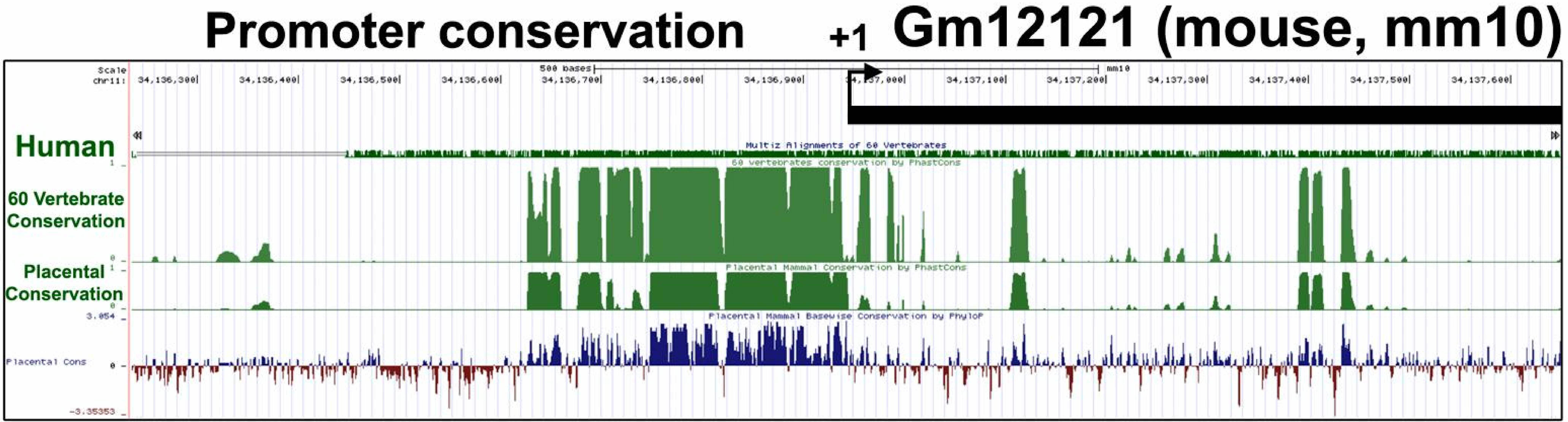

Supplementary Figure S5

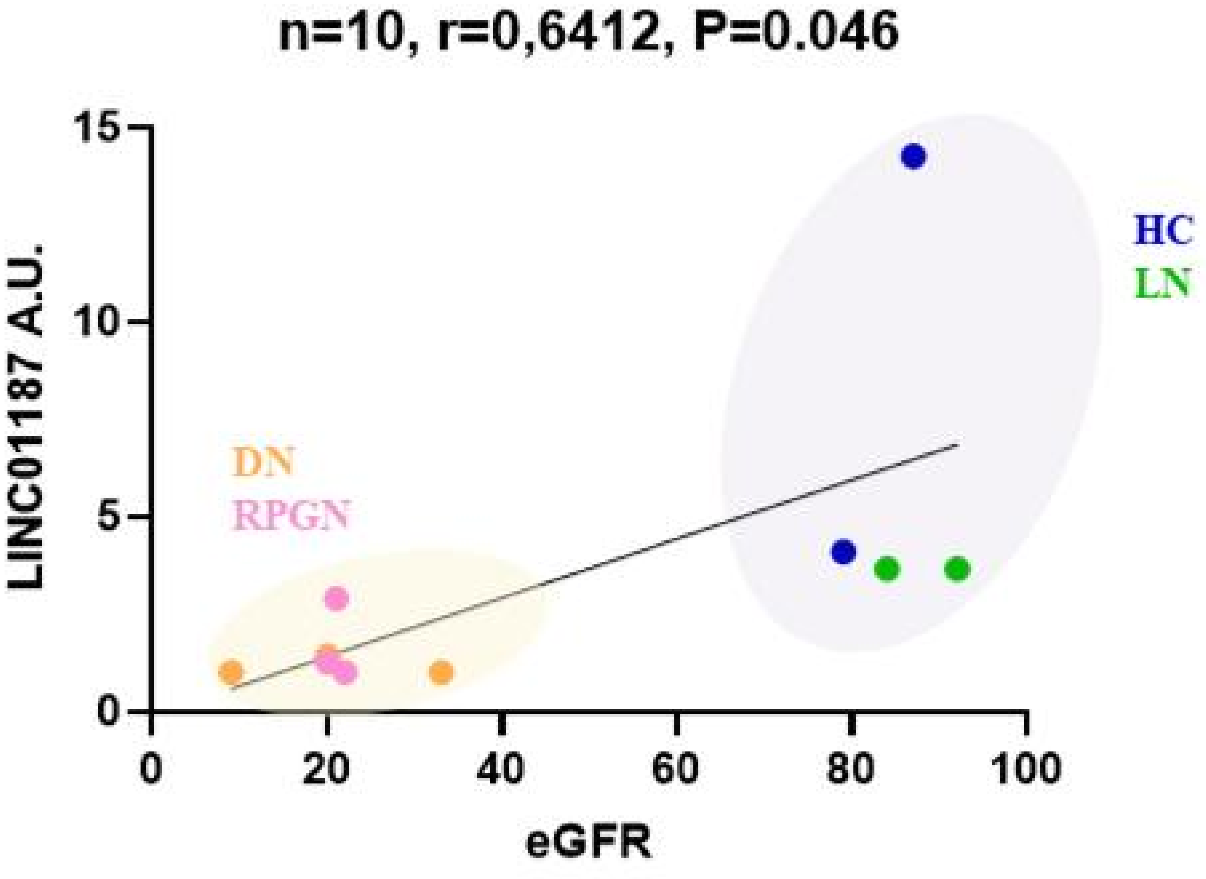

Supplement: Supplementary file 1 — Figures S1‐S5 [file JCMM-27-1192-s001.pdf]
